# Accumulation of Long-Chain Glycosphingolipids during Aging Is Prevented by Caloric Restriction

**DOI:** 10.1371/journal.pone.0020411

**Published:** 2011-06-08

**Authors:** María José Hernández-Corbacho, Russell W. Jenkins, Christopher J. Clarke, Yusuf A. Hannun, Lina M. Obeid, Ashley J. Snider, Leah J. Siskind

**Affiliations:** 1 Department of Medicine Medical University of South Carolina, Charleston, South Carolina, United States of America; 2 Department of Biochemistry and Molecular Biology, Medical University of South Carolina, Charleston, South Carolina, United States of America; 3 Ralph H. Johnson Veterans Affairs Medical Center, Charleston, South Carolina, United States of America; University of Geneva, Switzerland

## Abstract

**Background:**

Chronic kidney disease and end-stage renal disease are major causes of morbidity and mortality that are seen far more commonly in the aged population. Interestingly, kidney function declines during aging even in the absence of underlying renal disease. Declining renal function has been associated with age-related cellular damage and dysfunction with reports of increased levels of apoptosis, necrosis, and inflammation in the aged kidney. Bioactive sphingolipids have been shown to regulate these same cellular processes, and have also been suggested to play a role in aging and cellular senescence.

**Methodology/Principal Findings:**

We hypothesized that alterations in kidney sphingolipids play a role in the declining kidney function that occurs during aging. To begin to address this, the sphingolipid profile was measured in young (3 mo), middle aged (9 mo) and old (17 mo) C57BL/6 male mice. Interestingly, while modest changes in ceramides and sphingoid bases were evident in kidneys from older mice, the most dramatic elevations were seen in long-chain hexosylceramides (HexCer) and lactosylceramides (LacCer), with C14- and C16-lactosylceramides elevated as much as 8 and 12-fold, respectively. Increases in long-chain LacCers during aging are not exclusive to the kidney, as they also occur in the liver and brain. Importantly, caloric restriction, previously shown to prevent the declining kidney function seen in aging, inhibits accumulation of long-chain HexCer/LacCers and prevents the age-associated elevation of enzymes involved in their synthesis. Additionally, long-chain LacCers are also significantly elevated in human fibroblasts isolated from elderly individuals.

**Conclusion/Significance:**

This study demonstrates accumulation of the glycosphingolipids HexCer and LacCer in several different organs in rodents and humans during aging. In addition, data demonstrate that HexCer and LacCer metabolism is regulated by caloric restriction. Taken together, data suggest that HexCer/LacCers are important mediators of cellular processes fundamental to mammalian aging.

## Introduction

Many factors are thought to contribute to mammalian aging including changes in gene expression, mitochondrial dysfunction, oxidative stress, shortening of telomeres, and accumulation of advanced glycation end-products. These factors eventually lead to a decline in organ function and the ability to respond to physiological and pathophysiological stimuli. In the kidney, there is an age-associated progressive deterioration in renal function even in the absence of obvious renal disease [1]–[3]. Moreover, many changes occur in the kidney prior to a decline in kidney function, including increases in pro-inflammatory enzymes and cytokines [4], [5]. Consequently, elucidating the early changes that lead to a decline in kidney function is critical for the development of therapeutics aimed at preserving kidney function during aging.

With normal aging, a number of functional and structural changes occur in the kidney both in humans and in laboratory animals. These include decreases in renal blood flow and glomerular filtration rate, changes in tubular function that impair the ability to concentrate urine, glomerulosclerosis, and tubulointerstitial fibrosis [1]–[3]. Furthermore, as drug excretion is also reduced in the aged kidney, the pharmacokinetics and pharmacodynamics of many drugs used by the elderly are altered, making them more likely to suffer long-term consequences following acute kidney injury [1]–[3]. Ordinarily following acute kidney injury, damaged tubules are repopulated by proliferation of renal epithelial cells; however, this ability to repopulate is greatly diminished in the aged kidney. Thus, the aged kidney has increased exposure to renal stressors, enhanced susceptibility to injury, and decreased ability to repair itself following injury. As a result, the elderly have an increased incidence of renal disease than younger adults, including chronic kidney disease and end-stage renal disease.

Caloric restriction (CR), without a reduction in essential nutrients, is well known to extend the median and maximum lifespan of mammals and suppresses aging related diseases that shorten lifespan [6]–[8]. Importantly, CR also maintains kidney function during aging [9]–[11]. CR is thought preserve kidney function during aging via reduced levels of cellular processes such as apoptosis, necrosis, and inflammation which are all known to be elevated during renal aging [11]–[15]. Thus, understanding precise mechanisms by which CR protects kidney function during aging is highly valuable for the future development of novel therapeutics.

Sphingolipids represent a class of bioactive lipids that play important regulatory roles in apoptosis, necrosis, inflammation, insulin resistance and diabetes, all of which greatly impact kidney function and play large roles in kidney disease [16]–[24]. Ceramide is a sphingolipid that has been implicated in a variety of cell stress responses, but is also at the center of sphingolipid metabolism and is utilized for the synthesis of other bioactive sphingolipids, including sphingosine-1-phosphate (S1P), sphingosine (Sph), and the glycosphingolipids hexosylceramide (HexCer) and lactosylceramide (LacCer).

In recent years, evidence has begun to accumulate implicating ceramides and glycosphingolipids in a number of kidney pathologies such as acute renal failure, polycystic kidney disease, Fabry's kidney disease, renal cancer, and diabetic nephropathy [25]–[39]. As sphingolipid levels change during aging in the liver and brain [40], [41], we hypothesized that altered sphingolipid metabolism in the kidney plays an important role in kidney aging. To test this hypothesis, sphingolipids were measured in the kidneys from C57BL/6 male mice at different ages fed both *ad libitum* (AL) or a CR diet. Data revealed that long-chain HexCers and LacCers are significantly elevated during renal aging. These glycosphingolipids are also elevated during aging in the mouse brain and liver as well as in human fibroblasts obtained from elderly individuals. Importantly, CR effectively prevented the accumulation of long-chain HexCers and LacCers in the kidneys of aged mice. Taken together, these data suggest that HexCers and LacCers are important mediator of cellular processes fundamental to mammalian aging.

## Results

### HexCers and LacCers accumulate in the kidneys of aged mice

**Figure 1:**
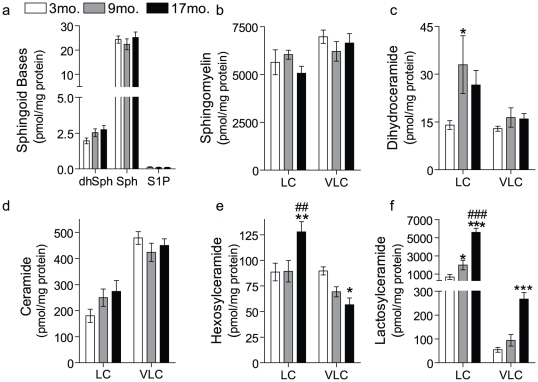
Renal sphingolipid levels in the kidneys of young, middle-aged, and older mice. The sphingolipid profile of kidney homogenates obtained from young (3 mo), middle-aged (9 mo) and older (17 mo) C57BL/6 male mice from the NIH calorically-restricted rodent colony. Sphingolipids were quantified by mass spectrometry and normalized to total protein. (a) sphingoid bases including sphingosine (Sph), dihydrosphingosine (dhSph) and sphingosine-1-phosphate (S1P), (b) Sphingomeylin, (c) dihydroceramide, (d) ceramide, (e) hexosylceramide, and (f) lactosylceramide where LC refers to the total long-chain species (C_14_–C_20_) species and VLC to the total very long-chain species (C_22_–C_26_). Data represent mean ± SEM; n = 6. * p<0.05, **p<0.01, and ***p<0.001, based on nonparametric one-way ANOVA; * indicates significance compared to 3 mo. mice, # indicates significance compared to the 9 mo. mice.

Increases in ceramide and sphingosine have been reported in the liver and brain of aged rats [41], [42] and were suggested to be a hallmark of the aging process. Thus, we hypothesized that similar changes in sphingolipid metabolism occur during kidney aging. To test this hypothesis, sphingolipids were quantified from kidney homogenates of C57BL/6 male mice 3, 9 and 17-months of age AL from the NIH calorically-restricted rodent colony. Surprisingly, results indicated that levels of the sphingoid bases such as sphingosine (Sph), dihydrosphingosine (dhSph) and sphingosine-1-phosphate (S1P) did not change significantly with age (Fig. 1a). Similarly, the levels of sphingomyelin (SM) were not significantly different between age groups (Fig. 1b). In contrast, dihydroceramide (dhCer) levels (Fig. 1c) were significantly elevated at 9 mo. but were not significantly elevated in the kidneys of 17 mo. mice. Similarly, long-chain ceramides also increased with age but not in a statistically significant manner (Fig. 1d). Likewise, there were no significant changes in the individual ceramide species (data not shown).

**Figure 2:**
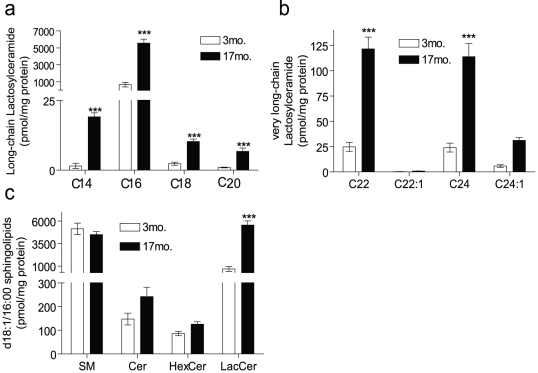
Abundance and levels of individual lactosylceramide species during aging in the kidney. Sphingolipids were measured in 3 mo. and 17 mo. old AL mice and the individual (a) long-chain (LC, C_14_–C_20_), (b) very-long chain (VLC, C_22_–C_26_) lactosylceramide species; and c) sphingolipids with C16 fatty acyl chains are shown. Data represent mean ± SEM; n = 6. ***p<0.001 according to a nonparametric one-way ANOVA.

As glycosphingolipids are highly abundant in the kidney and play a role in a variety of kidney diseases [28], [29], [31], [39], [43]–[46], we speculated that there could be age-associated differences in their levels; accordingly, this was investigated. As depicted in Figs. 1e and 1f, the most significant changes of all the sphingolipids measured were that of the simple glycosphingolipids, HexCer and LacCer. Notably, for HexCers, only the long-chain species were increased with aging, whereas very long-chain species were decreased (Fig. 1e). Moreover, within the long-chain species, C_16_-HexCer was the only species that accumulated during aging (Figure S1). In contrast, both long-chain and very long-chain species of LacCer were significantly elevated during aging (Fig. 1f). We determined whether specific LacCer species were preferentially elevated during aging. As is evident from Figs. 2a and 2b, all species of LacCer were significantly elevated with C_14_ and C_16_ species being the most significant. These elevations represent major changes in sphingolipid mass as in the aged kidney C_16_-LacCer is the most abundant of all of the classes of sphingolipids measured, including SM (Fig. 2c).

### The kidney, not the blood, is the source of Hexcers and LacCers during aging

**Figure 3:**
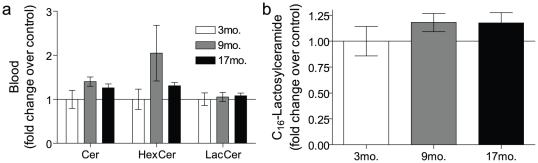
Hexosylceramides (HexCer) and lactosylceramides (LacCer) were not elevated in the blood during aging. Sphingolipids were measured in the 100 µL of whole blood obtained from 3, 9 and 17 mo. AL mice. Data are expressed as a fold-change over the 3 mo. animals for (a) total ceramides (Cer), HexCer and LacCer as well as (b) C_16_-LacCer. Data represent mean ± SEM; n = 6.

Sphingolipids in the blood are part of the circulating lipoprotein particles (VLDL, LDL, and HDL), are transported by serum albumin, and are present in blood cells and platelets [47]. As the blood is filtered by the kidneys, we reasoned that the blood could be the source of the renal accumulations of HexCer and LacCer during aging. To determine if this was the case, the blood sphingolipid profiles of the various mice age groups were measured. As can be seen, there were no significant increases in total ceramides, HexCers or LacCers during aging in the blood (Fig. 3a) and the individual species of ceramide and HexCer were also not significantly altered (data not shown). Furthermore, the major LacCer species present in the kidney, C_16_-LacCer, did not change in the blood during aging (Fig. 3b) and although there were elevations in some species of LacCer in the blood, such as C_14_, C_22_ and C_24_-LacCer, these were minor changes in mass and represent species with very low abundance in the kidney (data not shown). Finally, if blood was the source of the observed LacCer and HexCer elevations in the aged kidney, then there should be comparable elevations in these sphingolipids in other highly perfused organs such as the heart. However, no changes in either HexCer or LacCer in the heart were observed during aging (data not shown). Taken together, these results suggest that the glycosphingolipids were synthesized within the kidney during aging.

### HexCer and LacCer accumulations during aging were not restricted to the kidney, but occur in the brain and liver

**Figure 4:**
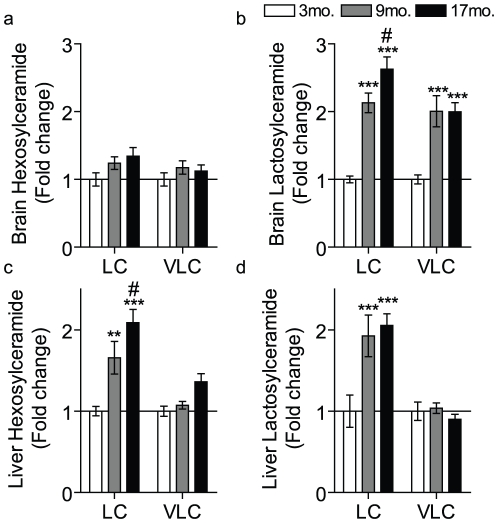
HexCers and LacCers are elevated in the brain and liver during aging. The sphingolipid profile was measured in brain (a and b) and liver (c and d) of 3, 9 and 17 mo. AL mice. Data were normalized to total protein and expressed as a fold-change over the 3 month mice. Data represent the mean ± SEM; n = 6. (a–d) LC refers to the total long-chain species (C_14_–C_20_) species and VLC to the total very long-chain species (C_22_–C_26_). * p<0.05, **p<0.01, and ***p<0.001 according to nonparametric one-way ANOVA. For a–d * indicates significance compared to the 3 mo. and # indicates significance compared to the 9 mo.

As elevations in sphingosine and ceramide have been documented in the brain and liver during aging [41], [42], we hypothesized that the HexCer and LacCer accumulations during aging are not restricted to the kidney. Accordingly, HexCer and LacCer species in the brain and liver of the mice described above were analyzed. Results showed that only the long-chain and very long-chain LacCers increased with age in brain whereas HexCers were unchanged (Fig 4a and b). In contrast, in the liver, both long-chain HexCers and long-chain LacCers were elevated during aging (Fig 4c and d). Overall, these data suggest that the changes in long-chain HexCer and LacCer are observed in many, but not all (e.g. heart), tissues.

### Caloric restriction decreases renal long-chain HexCers and long-chain LacCers

Increased reactive oxygen species (ROS) and levels of cellular processes such as inflammation, apoptosis, proliferation, and senescence occur during aging and have been proposed to shorten lifespan [48]–[50]. CR attenuates the levels of these processes during aging thereby extending lifespan and health span [11], [51], [52]. As glycosphingolipids such as HexCer and LacCer regulate these cellular processes *in vitro* [53]–[60], we hypothesized that CR is a negative regulator of HexCer/LacCer levels. To begin to address this hypothesis, we examined the impact of CR on renal sphingolipids in the absence of an additional factor such as aging. Sphingolipids were analyzed in kidney homogenates from C57BL/6 male mice obtained from the NIH NIA calorically-restricted rodent colony either fed AL or a CR diet. Following analysis, the renal sphingolipid profiles of the CR animals were compared to age-matched AL control animals. Very long-chain SM species were increased in mice on the CR diet (Figure S2). As SM is a highly abundant sphingolipid, this represents a substantial change in mass. Similar to the increase in very long-chain SM, mice fed the CR diet exhibited increases in very long-chain Cers (Figure S2). Interestingly very long-chain HexCers and very long-chain LacCers were decreased with CR. In addition, CR significantly reduced long-chain HexCers and LacCers (Figure S2), with the decrease in long-chain LacCers being the most significant. Thus, the CR diet profoundly shifted the sphingolipid profile by decreasing the long-chain Cers, HexCer, and LacCer and by increasing the very long-chain Cers and very long-chain SM. These results indicate that CR regulates sphingolipid metabolism in an opposite pattern to that observed during aging. Thus, CR is a negative regulator of renal HexCer/LacCer levels.

**Figure 5:**
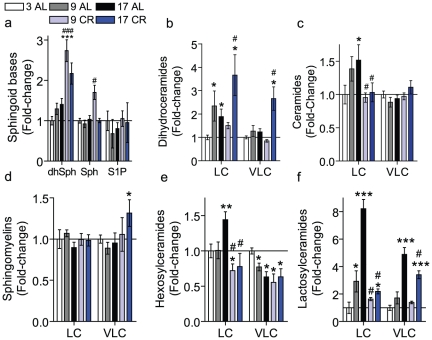
Impact of caloric restriction on renal LC-HexCers and LC-LacCers during aging. The sphingolipid profile was measured in the kidneys of 3, 9, or 17 mo. old mice fed either AL or on CR diet. Data were normalized to total protein, totals from the LC (C_14_–C_20_) and VLC (C_22_–C_26_) species calculated, and expressed as the fold-change of the 3 mo. mice. (A) sphingoid bases dihydrosphingosine (dhSph), sphingosine (Sph), and sphingosine-1-phosphate (S1P); (b) dihydroceramides; (c) ceramides; (d) sphingomyelins; (e) hexosylceramides; and (f) lactosylceramides. Data represent mean ± SEM; n = 5–6. Statistical analysis was performed using a rank-transformation followed by a two-way ANOVA with a Bonferroni post-test. * indicates significant difference compared to the 3 mo. AL mice, whereas # indicates a significant difference compared to the age-matched AL value. * p<0.05, **p<0.01, ***p<0.001.

To further understand the effects of CR on kidney sphingolipids during aging, data were expressed as a fold-change of the 3 mo. control mice so that both the variables of diet and age could be considered together. Sphingoid bases were elevated during aging in CR animals (Fig. 5a); specifically, dhSph was increased 3-fold in aged CR mice (Fig. 5a) and Sph was elevated almost 2-fold in the 9 mo. CR mice. S1P was not changed during aging in the CR mice. Both long-chain and very long-chain dihydroceramide species were elevated during aging in the CR animals to a much larger extent than in the AL fed animals (Fig. 5b). These increases in dihydroceramides in the aged CR mice did not translate to increases in long-chain Cers, as they were not elevated during aging in animals on a CR diet (Fig. 5c). Long-chain SM was elevated during aging in the CR animals, which represents a substantial change in mass as the long-chain SM are highly abundant in the kidney (Fig. 5d). Importantly, the elevations in long-chain HexCer and LacCer observed in AL animals during aging are not found in CR mice. Restriction of calories was sufficient to prevent accumulations in the long-chain HexCer and long-chain LacCer species during aging, but not the very long-chain species (Fig. 5e and f). Thus, these data suggest that long-chain glycosphingolipids are important mediators of biological processes fundamental to aging.

### Caloric restriction prevents the age-associated increase in neutral sphingomyelinase and long-chain ceramide synthase

**Figure 6:**
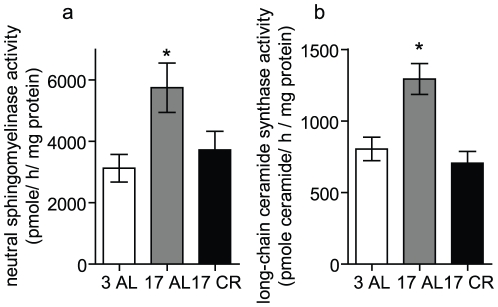
Neutral sphingomyelinase and long-chain ceramide synthase activities in kidney homogenates of young and old mice fed *ad libitum* and calorically restricted. Neutral sphingomyelinase (a) and long-chain ceramide synthase (b) activities were measured in kidney homogenates from the indicated aged mice (in months) fed *ad libitum* (AL) or on a calorically restricted (CR) regime as described in the materials and methods. Data represent mean ± SEM; n = 5–6. * p<0.05 as compared to the 3 AL control mice according to nonparametric one-way ANOVA.

The above data suggest that CR regulates enzymes responsible for the accumulation of HexCer and LacCer. During aging there is enhanced production of ceramides within the kidney, which is prevented by CR (Fig. 5). Ceramides are required substrates for the production of glycosphingolipids. Indeed, enzymes involved in ceramide synthesis are altered during aging and elevated liver neutral sphingomyelinase activity during aging is attenuated by CR [61], [62]. Thus, we hypothesized that HexCer and LacCer accumulations during aging occur as a result of enhanced substrate availability. To begin to address this hypothesis, we measured the activities of enzymes responsible for synthesis of ceramide in homogenates prepared from the kidneys of 3 and 17 month old mice fed AL as well as 17 month old mice on a CR diet. Both neutral sphingomyelinase (nSMase) and long-chain ceramide synthase (CerS) activities were elevated in the kidney during aging (Fig. 6a and b). Importantly, CR attenuated the age-associated increase in both nSMase and long-chain CerS activity (Figs. 6a and 6b). These data are consistent with the lipid data (Fig. 5) and suggest that CR prevents accumulation of HexCer and LacCer during aging, at least in part, by preventing the age-associated increase in nSMase and long-chain CerS activities.

### Long-chain HexCer and LacCer accumulate during aging in humans

**Figure 7:**
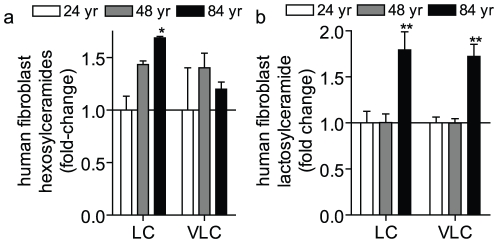
Long-chain HexCer and LacCer levels in aged human tissues. HexCer (a) and LacCer (b) were measured in commercially available (Coriel) human fibroblast cells isolated from 24, 48 and 84 year-old humans and grown in culture as described in the materials and methods. LC refers to the total long-chain species (C_14_–C_20_) species and VLC to the total very long-chain species (C_22_–C_26_). Data represent the mean ± SEM; n = 3. * p<0.05, **p<0.01, and ***p<0.001 according to nonparametric one-way ANOVA.

The above data implicate glycosphingolipids as important factors in mouse aging, particularly in the kidney, brain and liver. Thus, it became important to determine whether HexCer and LacCer accumulations during aging were also relevant to humans. For this, commercially available fibroblasts isolated from humans at 24, 48, and 84 years of age and grown in culture were utilized. As can be seen, long-chain HexCers were significantly higher in the fibroblasts of the 84 year old. This was also true for both the long-chain and very long-chain LacCers species (Fig. 7a and 7b). Notably, there were no statistically significant changes in any other sphingolipid species measured (data not shown). Taken together, this suggests that elevated levels of long-chain HexCer and LacCers are relevant biomarkers of aging in humans as well as in murine models.

## Discussion

In this study, we report elevations in the levels of the glycosphingolipids HexCers and LacCer during aging in the kidney, liver and brain of C57BL/6 male mice fed *ad libitum*. In addition, elevations in glycosphingolipids were also observed in human fibroblasts isolated from elderly individuals compared to younger individuals. Importantly, CR inhibited glycosphingolipid accumulation during aging in the kidneys. To our knowledge, this is the first study to describe alterations in kidney sphingolipids during aging. More importantly, this is the first report describing regulation of sphingolipid metabolism by an intervention known to attenuate aging. As CR is known to extend median and maximum lifespan of mammals, these data suggest that glycosphingolipid mediate declining renal function during aging.

Given the findings of this study and published work on renal glycosphingolipids, the results suggest the involvement of glycosphingolipids in the deterioration of renal function during aging. Glycosphingolipids such as HexCer and LacCer are highly abundant in the kidney and considerable evidence has implicated them in a variety of kidney pathologies [53]–[59]. For example, glycosphingolipid accumulation has been reported in models of polycystic kidney disease whereas preventing their accumulation with inhibitors of glucosylceramide synthase was shown to prevent the onset of this disease [29]. Furthermore, accumulation of glycosphingolipids also occurs in Fabry's kidney disease, glomerular nephritis, and in renal cancer [28], [35], [39], [43], [45], [63]–[65]. The current work demonstrates increased glycosphingolipid levels in the kidney during aging, thereby providing a possible and plausible explanation for the increased susceptibility and incidence of kidney disease seen in the elderly. Consistent with this theory, CR is well known to slow the aging process in a wide range of species including mammals, has been shown to maintain kidney function throughout aging and, here, was found to prevent the accumulation of HexCers and LacCers. Thus, therapeutics aimed at the regulation of glycosphingolipids may be useful for the maintenance of kidney function during aging, as well as being able to treat a variety of kidney diseases.

In addition to accumulation of glycosphingolipids, our data also show increased ceramide levels in the aged kidneys that is also prevented by CR, consistent with a profound alteration of sphingolipid metabolism during aging. Notably, these data are similar to previous reports demonstrating elevations in sphingosine and ceramide levels in aging livers of rats [41], [66]. In the brain, C_24_-galactosylceramide has been reported to accumulate in the cerebral cortex during aging [40]. Unlike Cutler et al., 2004, we do not detect statistically significant changes in C_24_-hexosylceramide in the aged brain, but rather only an accumulation of lactosylceramides. However, our results may differ than those previously reported [40] for several reasons, including measurement of hexosylceramides rather than specifically galactosylceramides, utilization of a different methods of sacrifice, use of different aged mice. Regardless, data presented here extend on the results of earlier studies by demonstrating elevated levels of LacCers in both liver and brain during aging.

The involvement of sphingolipids in aging is perhaps not surprising; indeed, there is considerable evidence linking sphingolipid genes with life span. For example, the longevity assurance gene (LAG1) in yeast was one of the first genes implicated in yeast aging [67] whose deletion was found to extend lifespan and was subsequently discovered to encode a ceramide synthase [68], [69], a key enzyme in the ceramide synthetic pathway. Furthermore, sphingolipid metabolism has been shown to play an important role in determining *Drosophila* lifespan [70], [71]. Breakdown of ceramide into sphingosine and fatty acids is catalyzed by the enzyme ceramidase. It was recently reported that the *Drosophila* alkaline ceramidase plays an important role in Drosophila development and longevity as mutation of alkaline ceramidase significantly increases the mean and maximum lifespan [71]. Finally, the ceramide transfer protein (CERT) regulates cellular ceramide levels by transporting ceramide from the ER to the Golgi where it is used specifically for SM synthesis. Notably, CERT mutant flies have enhanced oxidative damage and a dramatically reduced lifespan [70]. Taken together, these studies in model organisms such as yeast and *Drosophila* firmly establish that sphingolipids play important roles in determining lifespan and, by extension, are important regulators of the aging process.

Although a considerable increase in HexCers and LacCers was observed in aged kidneys in the present study, the mechanism by which this occurs is unclear. Given that ceramide levels were also increased and ceramide is a precursor of the complex sphingolipids, this implies enhanced synthesis. Consistent with this theory, neutral sphingomyelinase (nSMase) and long-chain ceramide synthase (CerS) activities are elevated in the kidney during aging, which are prevented by caloric restriction (Fig. 6a and b). Age-associated changes in the activity of a number of sphingolipid regulatory enzymes have been previously reported including nSMase2, ceramidase, and SM synthase [62]. Furthermore, during aging, there is elevated oxidative stress and free radical generation within the kidney that is reduced with CR. Importantly, nSMase2 is regulated by reactive oxygen species (ROS) [66], [72]–[75]. ROS-mediated activation of nSMase2 was implicated in the increased ceramide levels observed in the liver of aged rats [61], [66] and this was also attenuated by CR [61]. Moreover, glycosphingolipid synthesis is reported to be dependent on nSMase activity [60], [76]–[78]. Finally, elevated ROS levels have also been reported in many kidney diseases where glycosphingolipids are also known to play a role [79]–[83]. Taken together, this suggests that glycosphingolipid levels increase during aging owing to increased production of substrates, and this is prevented by CR. Certainly, our lipid data and our enzyme activity measurements are consistent with this possibility (Figs. 5 and 6). However, we cannot rule out the possibility that enhanced breakdown of complex glycosphingolipids could also contribute to accumulations in HexCer/LacCer. Alternatively, enhanced production of HexCer and LacCer could also lead to elevated levels of complex glycosphingolipids in the aged kidney, including globosides and gangliosides that are highly abundant in the kidney and play a role in numerous kidney diseases [84]. Thus, studies are underway to both evaluate the contribution of the different sphingolipid enzymes to the accumulation of HexCer and LacCer during aging as well as determine if altered metabolism of complex glycosphingolipids plays a role in renal aging.

Although the results raise the possibility that glycosphingolipids may play important roles in renal aging, the mechanism(s) by which glycosphingolipid accumulation adversely affects the kidney are unclear. Both HexCer and LacCer are well established regulators of biological processes such as cell proliferation, apoptosis and inflammation [53]–[59]. Consequently, alterations in any (or all) of these processes may contribute to age-related pathology. However, to begin to address the mechanisms of glycosphingolipid action more fully, it will first be important to understand the location of accumulated glycosphingolipids within the kidney. While the current data confirm that increased HexCer and LacCer are synthesized within the kidney rather than delivered by the blood, there are many different cell types within the kidney that could act as the source. Indeed, identifying the location within the kidney in which HexCer and LacCer accumulate during aging will offer some insight into their relevant pathologies.

In summary, this study demonstrates accumulation of the glycosphingolipids HexCer and LacCer during kidney aging that is prevented by CR. This implicates the glycosphingolipids as important mediators of kidney aging and, indeed they regulate processes fundamental to aging. As renal disease represents a significant cause of morbidity and mortality in the elderly population, these data open a new avenue of potential therapeutic intervention. Indeed, as inhibitors of glycosphingolipid synthesis are well tolerated in rodents and are currently being utilized for treatment of Gaucher disease patients, the potential for translation to humans is considerable.

## Materials and Methods

### Materials

BCA Protein Determination Kit was from Pierce (Rockford, IL).Phosphatase inhibitor cocktails were from Sigma (St. Louis, MO) and protease inhibitor tablets were from Roche Diagnostics (Indianapolis, IN). *Choline*-*methyl*-^14^C]sphingomyelin was synthesized by and obtained from MUSC Lipidomics Core Facility. C17-sphingosine, porcine brain sphingomyelin, and C_16_-fatty acyl CoA were obtained from Avanti Polar Lipids (Alabaster, AL).

### Cell Culture

Human dermal fibroblasts from 24- (AG11732), 48- (AG11793), and 84-year old (AG11725) females were obtained from the Coriell Cell Repository. Cells were maintained in high-glucose DMEM supplemented with 10% (v/v) fetal bovine serum without antibiotics. Cells were used from passage 10–16. For sphingolipid measurements, adherent cells were washed twice with cold PBS and lysed in buffer containing 1% Triton X-100, 50 mM Tris (pH 7.4), 0.15 M NaCl, 1.0 mM EDTA, supplemented with protease and phosphatase inhibitors. Cellular homogenates (0.1–1.0 mg) were frozen at −80°C until submission for sphingolipid analysis.

### Animals and Diets

Male C57BL/6 mice were purchased from the National Institute of Health National Institute of Aging (NIA) aged rodent colonies (Bethesda, MD, USA). Animals from 3 different ages were used in this study: 3, 9 and 17-month old. All mice were maintained on a 12-hour light/12-hour dark cycle and provided food and water AL for 3 months. At 14 weeks of age, they were divided into 2 groups, singly housed, and either kept on the AL diet or initiated on a CR diet. For each group at least 5 total animals were utilized. The final CR diet consisted of a 40% reduction in total calories without a reduction in essential nutrients and was initiated in a stepwise fashion. All animal procedures were approved by the Medical University of South Carolina (MUSC) Institutional Animal Care and Use Committee (protocol approval #2760) and followed the guidelines of the American Veterinary Medical Association.

### Kidney, Plasma, Liver and Brain Collection and Homogenization

Seven to ten days after their arrival, animals were sacrificed and plasma collected. The kidney, liver and brain were rapidly removed and weighed. The organs were snap frozen in liquid nitrogen and stored at −80C. Tissue samples were excised from each frozen organ and then homogenized, using a Tissue-Tearor (Biospec Products, Inc), in 20 mM Tris-HCl (pH 7.4) containing protease inhibitors.

### Quantification of sphingolipids

1 mg of protein from tissue or 0.1–1.0 mg protein from cellular homogenate was utilized for quantification of sphingolipids species performed by the Lipidomics Shared Resource Facility at the Medical University of South Carolina (MUSC) HPLC/MS as previously described [85]. Data was normalized to total protein as well as total lipid phosphate.

### Neutral sphingomyelinase activity

Neutral sphingomyelinase (nSMase) activity was assessed *in vitro* using [*choline*-*methyl*-^14^C] sphingomyelin as described previously [86]. Briefly and with modifications, kidney homogenates were diluted in lysis buffer (0.2% Triton X-100, 50 mM Tris-HCl-pH 7.4, 1.0 mM EDTA with phosphatase and protease inhibitors). 100 µL of reaction mixture was added containing 100 µM, 1×10^5^ cpm of [*choline*-*methyl*-^14^C] sphingomyelin (synthesized by and obtained from the MUSC Lipidiomics Core Facility) presented in micelles containing 0.2% Triton X-100 in 50 mM Tris-HCl (pH 7.4) and 0.1 mM ZnCl_2_. After 30 min at 37°C, the reaction was terminated by adding 1.5 mL of chloroform/methanol (2∶1, v/v) followed by addition of 0.4 mL of water. Samples were then vortexed and centrifuged at 2,000× g (5 min, room temperature) to separate phases. Aliquots (800 µL) of the upper (aqueous) phase were used for liquid scintillation counting. The activity was expressed as pmol/mg protein/h. 25 µg of kidney homogenate was used for nSMase activity measurements, as this was determined to be within the linear range for enzyme activity.

### Long-chain ceramide synthase activity measurements

Ceramide synthase activity was measured as previously described [87], [88]. Briefly and with modifications, 50 µg kidney homogenate was utilized as this was determined to be within the linear range for enzyme activity. A reaction mix (100 µL final volume) containing 15 µM C_17_ sphingosine and 50 µM C_16_ fatty acyl-CoA in 25 mM potassium phosphate buffer (pH 7.4) was pre-warmed at 37°C for 5 min. The enzyme reaction was initiated via addition of 50 µg kidney homogenate and after 15 min at 37°C was terminated via the addition of 2 mL of extraction solvent containing ethyl acetate/2-propanol/water (60/30/10 v/v/v) supplemented with internal standard for EI/LC/MS analysis. Lipids were extracted twice, dried under a stream of nitrogen, and resuspended into 150 µL 1 mM NH_4_COOH in 0.2% HCOOH in methanol and analyzed by EI/LC/MS by the MUSC Lipidomics Core Facility.

### Statistical Analysis

For statistical analysis an either one-way or two-way ANOVA was performed with a Bonferroni post-test. * indicates a statistically significant difference between the indicated value and the 3 mo. controls, # indicates a statistically significant difference between 9 mo. and 17 mo. animals. * p<0.05, **p<0.01, ***p<0.001.

## Supporting Information

Figure S1 **Levels of individual hexosylceramide species during aging in the kidney.** Hexosylceramides were measured in 3 mo. and 17 mo. old AL mice and the individual (a) long-chain (LC, C_14_–C_20_) and (b) very-long chain (VLC, C_22_–C_26_) species shown. Data represent mean ± SEM; n = 6. ***p<0.001 according to a nonparametric one-way ANOVA.
(PDF)

Figure S2 **Caloric restriction regulation of renal sphingolipid levels.** The sphingolipid profile was measured in 9 and 17 month old CR mice and age-matched littermate AL mice all obtained from the NIH NIA calorically-restricted mouse colony. Lipid data were normalized to total protein and the mean value calculated for each group. Data are expressed as a Log_2_ of the ratio of the CR mean to the age-matched littermate AL mean for (a) sphingomyelin, (b) ceramide, (c) hexosylceramide, and (d) lactosylceramide. LC refers to long-chain species (C_14_–C_20_) species and VLC to the very long-chain species (C_22_–C_26_). n = 5–6.
(PDF)
