# Supplementary material for: Accumulation of Long-Chain Glycosphingolipids during Aging Is Prevented by Caloric Restriction
Source: PLoS One. 2011 Jun 8;6(6):e20411. doi: 10.1371/journal.pone.0020411 (PMC3110726; doi:10.1371/journal.pone.0020411)
Supplement: Figure S1 — Levels of individual hexosylceramide species during aging in the kidney. Hexosylceramides were measured in 3 mo. and 17 mo. old AL mice and the individual (a) long-chain (LC, C14–C20) and (b) very-long chain (VLC, C22–C26) species shown. Data represent mean ± SEM; n = 6. ***p<0.001 according to a nonparametric one-way ANOVA. (PDF) [file pone.0020411.s001.pdf]

SUPPORTING INFORMATION S1:

S1 Figure Legend: Levels of individual hexosylceramide species during aging in the kidney. Hexosylceramides were measured in 3 mo. and 17 mo. old AL mice and the individual (a) long-chain (LC, C_{14}-C_{20}) and (b) very-long chain (VLC, C_{22}-C_{26}) species shown. Data represent mean ± SEM; n=6. ***p< 0.001 according to a nonparametric one-way ANOVA.

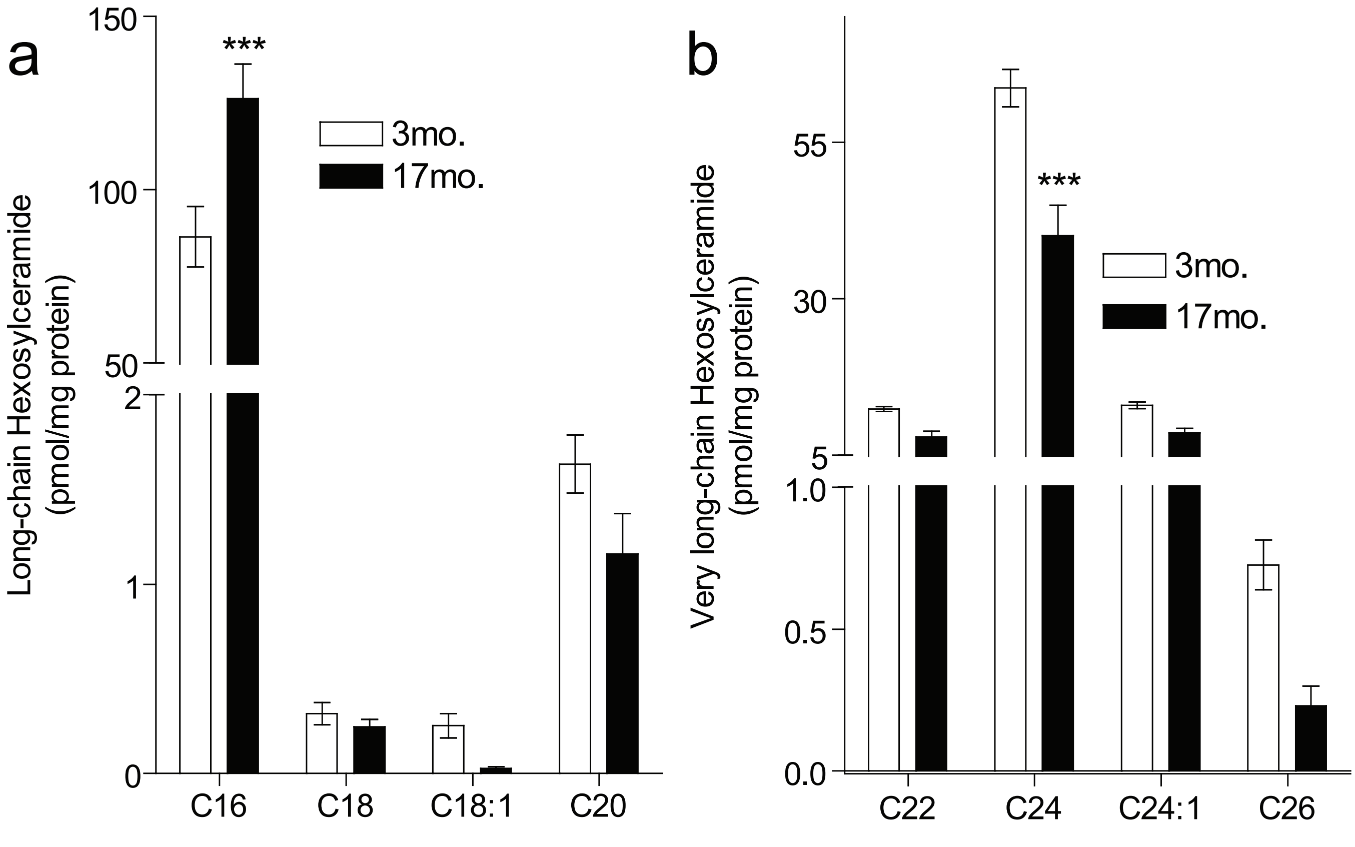

Supplemental Figure 1
