# Supplementary material for: Accumulation of Long-Chain Glycosphingolipids during Aging Is Prevented by Caloric Restriction
Source: PLoS One. 2011 Jun 8;6(6):e20411. doi: 10.1371/journal.pone.0020411 (PMC3110726; doi:10.1371/journal.pone.0020411)
Supplement: Figure S2 — Caloric restriction regulation of renal sphingolipid levels. The sphingolipid profile was measured in 9 and 17 month old CR mice and age-matched littermate AL mice all obtained from the NIH NIA calorically-restricted mouse colony. Lipid data were normalized to total protein and the mean value calculated for each group. Data are expressed as a Log2 of the ratio of the CR mean to the age-matched littermate AL mean for (a) sphingomyelin, (b) ceramide, (c) hexosylceramide, and (d) lactosylceramide. LC refers to long-chain species (C14–C20) species and VLC to the very long-chain species (C22–C26). n = 5–6. (PDF) [file pone.0020411.s002.pdf]

SUPPORTING INFORMATION S2:

S2 Figure Legend: Caloric restriction regulation of renal sphingolipid levels. The sphingolipid profile was measured in 9 and 17 month old CR mice and age-matched littermate AL mice all obtained from the NIH NIA calorically-restricted mouse colony. Lipid data were normalized to total protein and the mean value calculated for each group. Data are expressed as a Log_2 of the ratio of the CR mean to the age-matched littermate AL mean for (a) sphingomyelin, (b) ceramide, (c) hexosylceramide, and (d) lactosylceramide. LC refers to long-chain species (C_{14}-C_{20}) species and VLC to the very long-chain species (C_{22}-C_{26}). n = 5-6.

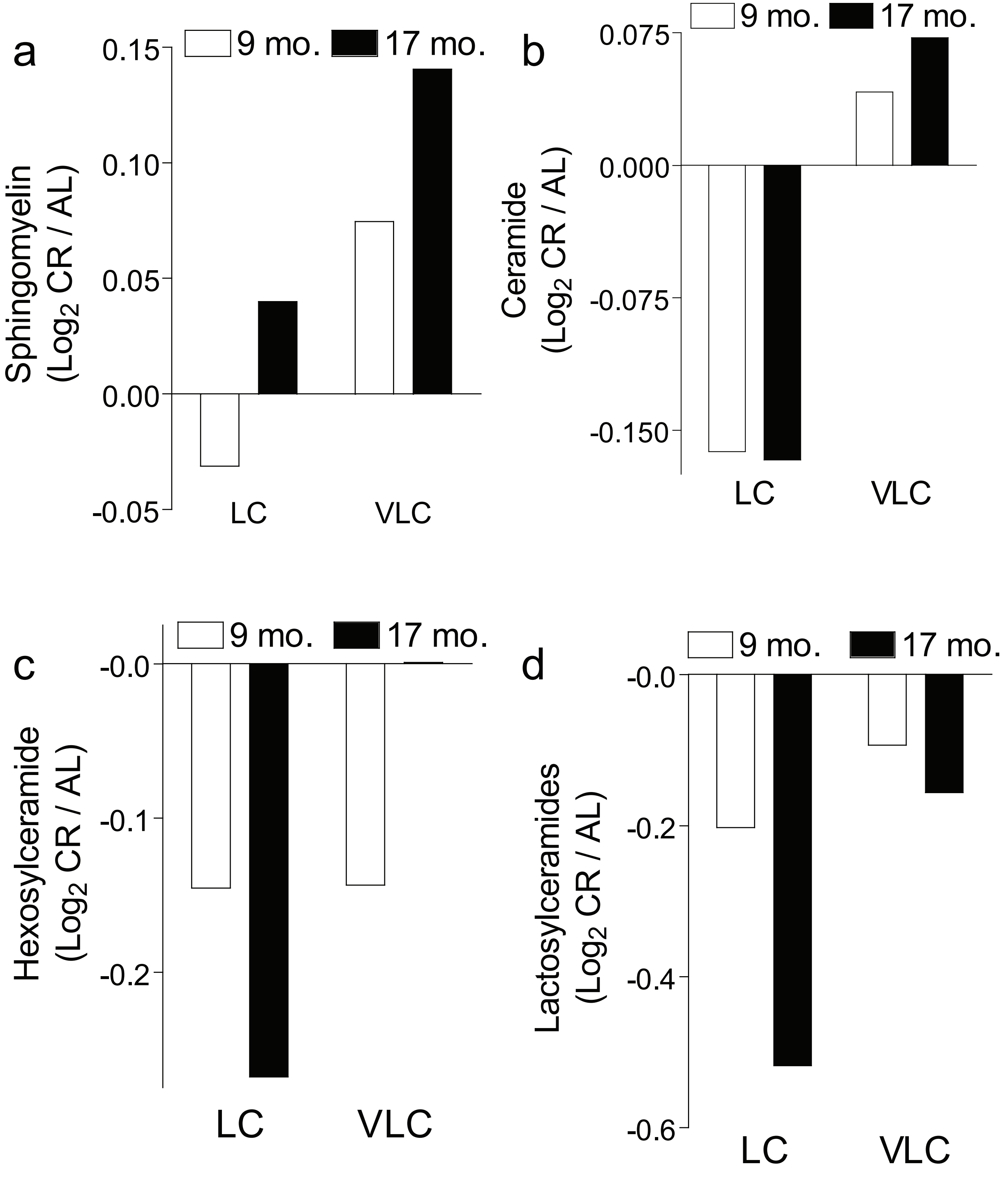

Supplemental Figure 2
